# Targeting the Hepatic Microenvironment to Improve Ischemia/Reperfusion Injury: New Insights into the Immune and Metabolic Compartments

**DOI:** 10.14336/AD.2022.0109

**Published:** 2022-07-11

**Authors:** Fengqiang Gao, Xun Qiu, Kai Wang, Chuxiao Shao, Wenjian Jin, Zhen Zhang, Xiao Xu

**Affiliations:** ^1^Department of Hepatobiliary and Pancreatic Surgery, The Center for Integrated Oncology and Precision Medicine, Affiliated Hangzhou First People’s Hospital, Zhejiang University School of Medicine, Hangzhou, China.; ^2^Zhejiang University Cancer Center, Hangzhou, China.; ^3^Department of Hepatobiliary and Pancreatic Surgery, The First Affiliated Hospital, Zhejiang University School of Medicine, Hangzhou, China.; ^4^NHC Key Laboratory of Combined Multi-organ Transplantation, Hangzhou, China.; ^5^Institute of Organ Transplantation, Zhejiang University, Hangzhou, China.; ^6^Zhejiang University School of Medicine, Hangzhou, China.; ^7^Department of Hepatobiliary and Pancreatic Surgery, Affiliated Lishui Hospital, Zhejiang University School of Medicine, Lishui, China.; ^8^Department of Hepatobiliary Surgery, the Third Affiliated Hospital of Soochow University, Changzhou, China

**Keywords:** hepatic microenvironment, ischemia/reperfusion injury, immune cell, metabolic compartment, inflammatory response, therapeutic strategies

## Abstract

Hepatic ischemia/reperfusion injury (IRI) is mainly characterized by high activation of immune inflammatory responses and metabolic responses. Understanding the molecular and metabolic mechanisms underlying development of hepatic IRI is critical for developing effective therapies for hepatic IRI. Recent advances in research have improved our understanding of the pathogenesis of IRI. During IRI, hepatocyte injury and inflammatory responses are mediated by crosstalk between the immune cells and metabolic components. This crosstalk can be targeted to treat or reverse hepatic IRI. Thus, a deep understanding of hepatic microenvironment, especially the immune and metabolic responses, can reveal new therapeutic opportunities for hepatic IRI. In this review, we describe important cells in the liver microenvironment (especially non-parenchymal cells) that regulate immune inflammatory responses. The role of metabolic components in the diagnosis and prevention of hepatic IRI are discussed. Furthermore, recent updated therapeutic strategies based on the hepatic microenvironment, including immune cells and metabolic components, are highlighted.

## 1. Introduction

Ischemia/reperfusion injury (IRI), resulting from ischemic insult and subsequent blood reperfusion, occurs in all aerobic cells that require mitochondrial oxidative phosphorylation for energy provision [1]. IRI is caused by trauma, shock, ischemic stroke, thrombolysis, coronary disease intervention and major surgeries [2-4]. Two major types of liver IRI have been reported, including warm injury and cold injury. Warm injury occurs during shock, trauma, respiratory failure, bleeding, heart failure, and prolonged surgical liver resection due to impaired blood perfusion [5,6]. Cold injury mainly occurs during liver transplantation due to *ex vivo* cold-preservation of the donor organ, and the subsequent warm reperfusion to the implanted organ [7].

The degree of hepatic IRI is dependent on duration and type of ischemia, as well as the condition of the liver [8,9]. In a clinical trial, cirrhotic or chronic liver injury showed worse tolerance to IR insult [10]. Evidence from preclinical experiments also support the standpoint that the background of hepatic microenvironment significantly determines the tolerance of IR [11].

The development of hepatic IRI is a complex process involving several factors such as metabolic disorders and inflammatory responses [12]. Considerable studies have investigated the molecular mechanisms of hepatic IRI, especially roles of Kupffer cells (KCs) in the generation of reactive oxygen species (ROS) and regulation of inducible nitric oxide synthase [13]. However, few studies have explored the impact of metabolic components on the development and progression of IRI. Furthermore, the key role of immune and metabolic compartments in hepatic microenvironment has not been systematically reported yet. In this review, the pathological changes associated with hepatic IRI are stated as well as the role of hepatic microenvironment (especially non-parenchymal cells). The immune responses and metabolic compartments involved in the process of IRI are discussed. Also, current therapeutic strategies for IRI are presented. Finally, the clinical translation potential of the findings from basic research are reviewed.

## 2. Pathophysiological alterations of hepatic IRI

**Figure 1.**
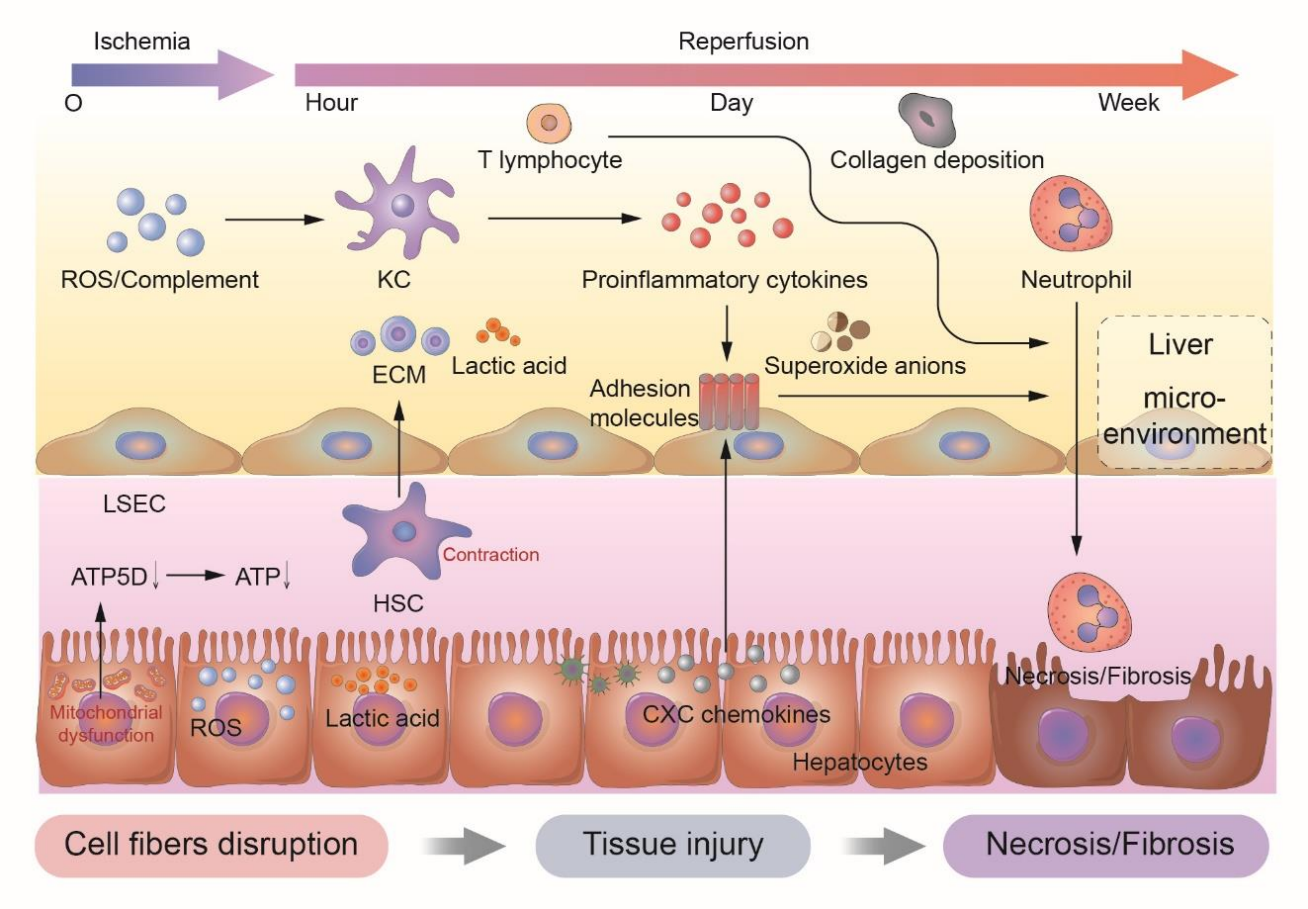
**The regulation of liver microenvironment during different periods of hepatic ischemia/reperfusion injury**. A summary of the immune components of liver microenvironment during the continuous phase of ischemia/reperfusion injury is manifested. LSEC, liver sinusoidal endothelial cell; HSC, hepatic stellate cell; KC, kupffer cell; ROS, reactive oxygen species; ECM, extracellular matrix.

Originally, IRI was first described by Jennings in 1960 as a phenomenon caused by hypoxia and accentuated by restoration of oxygen into tissues [14]. Development of hepatic IRI has two interrelated phases: ischemic insult and inflammation-mediated reperfusion injury (Fig. 1).

During liver transplantation, early allograft dysfunction (EAD) and primary non-function (PNF) have been associated with high rate of mortality and often due to perioperative IRI [15,16]. Numerous studies have demonstrated that IRI is one of the most important factors leading to EAD which has an incidence up to 43.7% in patients with IRI [17,18]. Hepatic EAD and failure of remnant liver increase the shortage of liver donors [19]. More importantly, the two factors may complicate post-transplant patient care, leading to poor liver transplantation (LT) outcomes. To improve outcomes, the specific mechanisms leading to hepatic IRI, and more targeted therapies should be explored.

**Figure 2.**
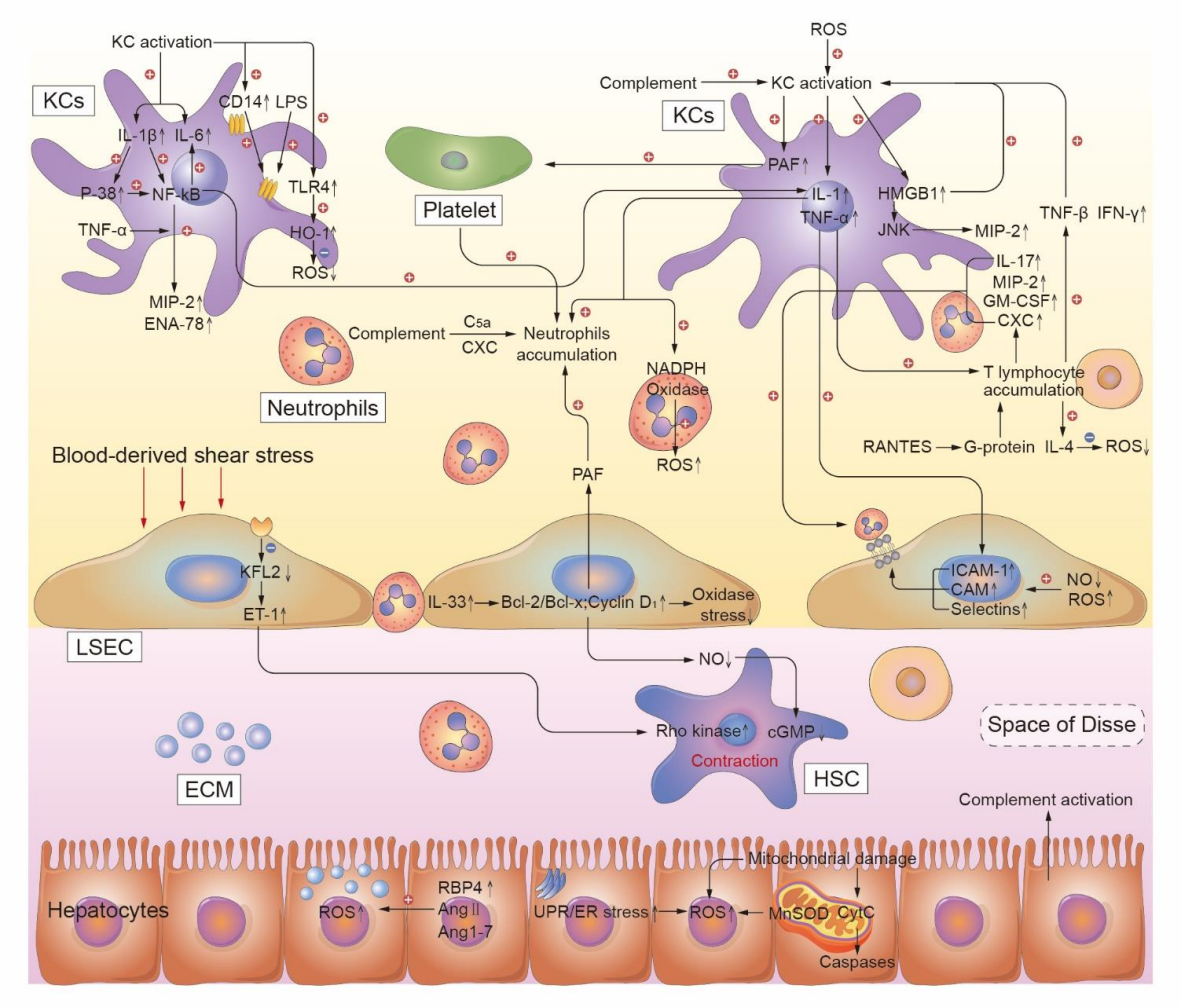
**The regulation of liver microenvironment components, including hepatic parenchymal cells, hepatic non-parenchymal cells (hepatic stellate cells, Kupffer cells, sinusoidal endothelial cells, neutrophils and lymphocytes), and extracellular matrix, during hepatic ischemia/reperfusion injury**. A summary of the specific molecular mechanisms regulating hepatocytes and interactions in the liver microenvironment are shown. LSEC, liver sinusoidal endothelial cell; HSC, hepatic stellate cell; KC, Kupffer cell; ROS, reactive oxygen species; ECM, extracellular matrix; IL-1, interleukin 1; IL-6, interleukin 6; IL-17, interleukin 17; IL-33, interleukin 33; IL-1β, interleukin 1β; HMGB1, high-mobility group box 1; IAC, inflammation associated cytokine; TNF-α, tumor necrosis factor α; PAF, platelet activating factor; MIP-2, macrophage inflammatory protein 2; ENA-78, epithelial neutrophil activating protein 78; NF-κB, nuclear factor κB; TLR4, Toll like receptor 4; LPS, lipopolysaccharide; HO-1, heme oxygenase-1; RANTES, regulated upon activation normal T cell expressed and secreted factor; VEGF, vascular endothelial growth factor; IFN-γ, interferon γ; GM-CSF, granulocyte-macrophage colony-stimulating factor; ICAM-1, intercellular adhesion molecule-1; Bcl-2/Bcl-x, B cell lymphoma 2/x; TXA2, thromboxane; KFL2, kruppel like transcription factor 2; ET-1, endothelin 1; JNK, N terminal kinase; CAM, cell adhesion molecule; cGMP, cyclic guanosine monophosphate; RBP4, retinol binding protein; Ang 1-7, angiotensin 1-7; Ang Ⅱ, angiotensin Ⅱ; MnSOD, manganese containing superoxide dismutase; CytC, cytochrome C.

The ischemia phase, which initiates reperfusion injury, is characterized by various factors involved in the inflammatory reactions. During this phase, vascular closure or obstruction decrease the expression of adenosis triphosphate synthase subunit delta (ATP5D) of the respiratory chain in mitochondria, thereby compromising ATP synthesis [20-22]. This is also accompanied by aggregation of lactic acid and ketone bodies in hepatic cells, causing metabolic acidosis [23].

The process of reperfusion has two phases. During the initial phase, KCs (liver-resident macrophages) are activated to induce oxidative stress. In the later period, 6-24 hours after reperfusion, numerous hepatic non-parenchymal cells are activated or accumulated to release inflammatory mediators, cytokines and complements [24,25]. Hypoxanthine oxidase catalyzes the breakdown of hypoxanthine to form water and oxygen, thus releasing ROS [26]. ROS generated by O_2_ reintroduction into ischemic tissues leads to severe liver damage. The mitochondria are important sources of ROS generated in the liver cells [27]. So, the maintenance of mitochondrial viability is important to the treatment of IRI. More importantly, it has been reported that AMP-activated protein kinase (AMPK) and protein kinase C (PKC) are activated by excessive AMP, resulting in the translocation of reduced form of nicotinamide-adenine dinucleotide phosphate (NADPH) subunits p67 and p47 from the cytosol to membrane, where they activate membrane subunit p91 and NADPH oxidase in turn [28]. Activation of NADPH oxidase leads to the production of large amounts of superoxide anions, which aggravate hepatic damage, leading to organ failure.

## 3. Role of age in hepatic IRI

In recent years, the percentage of liver grafts obtained from aged donors (over 70 years old) has been on the increase. This has led to the development of strategies to prevent IRI in aged individuals receiving liver transplantation. Accumulating experimental evidence has indicated that the aging process of liver has 3 dominant processes: enhancement of inflammatory response, impairment of intracellular energy metabolism and alteration of autophagy [29].

It has been reported that enhancing intercellular adenosine triphosphate (ATP) levels through glucose administration has effectively mitigated liver IRI [30]. In addition, application of pentoxifylline to inhibit the activation of TNF-α and melatonin to increase nitric oxide (NO) formation alleviated liver damage in aged individuals [31]. As for the autophagy, aging contributes to autophagy impairment, which renders aged livers susceptible to IRI. Lithium, as an autophagy inducer, has been shown to be effective to restore the reduced tolerance to liver IRI [32].

## 4. Hepatic microenvironment

The hepatic microenvironment comprises hepatic parenchymal cells, hepatic non-parenchymal cells (hepatic stellate cells, macrophages, sinusoidal endothelial cells, neutrophils, and lymphocytes), extracellular matrix, and nervous systems. Both parenchymal and non-parenchymal cells in the microenvironment modulate IRI progression, especially non-parenchymal cells [33-35]. Therefore, the characteristics of non-parenchymal cells (Fig. 2) will be discussed in more details in the ensuing sections.

### 4.1 Hepatic stellate cells

Hepatic stellate cells (HSCs), as the major components of the non-parenchymal cells in the liver, have important physiological and pathological roles on intrahepatic Disse space, which is an area between hepatocytes and sinusoids. HSCs can participate in the regulation of blood flow with extending cytoplasmic processes around hepatic sinusoids. Once activated, HSCs transform into myofibroblast-like cells, which express proteins like myosin and α-smooth muscle actin (α-SMA), allowing them to contract [36,37].

Endothelin (ET) has been reported to regulate hepatic sinus blood flow through HSCs [38]. Recent clinical trials have shown that HSCs are the effector of sevoflurane, which decrease ROS and hydrogen peroxide (H_2_O_2_) production, and hepatocytic apoptosis [39]. These effects are produced by suppressing the expression of BCL2-associated X (Bax) and elevating B cell lymphoma 2 (Bcl-2) levels[40]. In mouse model of IRI, Xu *et al*. showed that sevoflurane can inhibit the expression of high mobility group box 1 (HMGB1) to up-regulate microRNA (miR)-142 [41]. Adoptive HSCs confer protection against IRI by inducing newborn inducible regulatory T cells (iTregs) and increasing the stability of natural regulatory T cells (nTregs) [42]. Also, FGF10, belongs to the FGF subfamily, shown to be predominantly secreted by HSCs in vitro experiments [43]. In the early phase of IRI, overexpression of FGF10 alleviated liver dysfunction through the activation of phosphatidy-linositol-3-kinase (PI3K)/AKT/nuclear factor-erythroid 2-related factor 2 (NRF2) pathways. This protective role was abolished by NRF2 knockout in mice. Elsewhere, it was found that FGF10 overexpression also increased hepatocyte proliferation in the late phase of IRI [44,45].

The proliferation of HSCs at the boundaries of necrotic liver regions improves hepatic repair and regeneration [46]. Classical theory stipulates that those fibrotic livers are at a higher risk of developing IRI. However, fibrotic livers have an enhanced repair capacity compared with normal liver [47]. HSCs also can regulate the activity and viability of progenitor cells, adjacent hepatocytes, and Ly6C^lo^ macrophages, to promote hepatic recovery after IRI [48,49].

### 4.2 Macrophages

Liver injury provokes the activation of Kupffer cells (KCs), infiltration of circulating macrophages, and employment of peritoneal macrophages. Different subpopulations of macrophages have varying roles in the development of liver IRI [50]. During liver injury, majority of KCs are rapidly activated by ROS, releasing HMGB1 and inflammation-associated cytokines (IAC) like interleukin 1 (IL-1), interleukin 6 (IL-6), interleukin 1β (IL-1β), TNF-α and platelet-activating factor (PAF) [51,52]. In turn, ROS can cause mitochondrial damage, resulting in the leakage of mitochondrial deoxyribo-nucleic acid (mtDNA) into the cytosol [53]. Then the mtDNA can be recognized by DNA sensor cyclic GMP-AMP synthase (cGAS), and can activate stimulator of interferon genes (STING), leading to impaired innate immune response [54]. A previous animal study showed that increased mtDNA induced STING activation in macrophages, which triggered a more severe immune response accompanied by upregulating of IL-6, IL-18, IL-1β, and TNF-α levels via the STING-NLRP3 pathway [55].

A proportion of KCs are activated by IFN-γ, which is produced by CD4^+^ T-cells and natural killer T-cells [56]. Indeed, TNF-α and IL-1 are among the most important cytokines contributing to the development of hepatic IRI. IL-1 can stimulate the release of ROS from neutrophils, thereby amplifying TNF-α production [57]. TNF-α also provokes the expression of P-selectin in liver sinusoidal endothelial cells (LSECs), hence contribute to neutrophil recruitment [58]. In addition, TNF-α has been reported to enhance the release of other factors like macrophage inflammatory protein-2 (MIP-2), epithelial neutrophil activating protein-78 (ENA-78), cytokine-induced neutrophil chemoattractant-1 (CINC), and various CXC motif chemokines [59], which significantly enhance neutrophils infiltration.

Recent studies have demonstrated that the role of nuclear factor κB (NF-κB) varies across different cell types [51]. In the liver, NF-κB mainly promotes the expression of TNF-α and IL-6, contributing to inflammatory responses [60]. Resting-state KCs express a small amount of CD14 receptor and Toll-like receptor 4 (TLR4). However, it has been shown that CD14 receptor and TLR4 are upregulated in response to hepatic IRI. CD14, a receptor for complexes of lipopolysaccharide (LPS) and LPS binding protein, activates TLR4 upon binding to LPS, which leads to inflammatory responses and oxidative stress [61,62]. Moreover, the expression of transmembrane G protein-coupled bile acid receptor (TGR5) is highly observed in KCs. Yang *et al.* [63] found that TGR5 mitigated the increase in TLR4-NF-κB and reduced caspase 8 activation after IRI. In addition, TGR5 has been revealed to attenuate liver damage following IRI through the Keap 1-NRF2 pathway, as evidence by serum ALT and AST tests and cytokines expression [64]. Jin *et al.* [65] found that the activation of farnesoid X receptor (FXR) led to upregulation of small heterodimer partner (SHP) in KCs, which decreased the proinflammation injury but increased expression levels of anti-inflammatory gene expression following TLR stimulation.

The activation status of KCs correlates with the aggregation of platelets [66] and over 50% of platelets adhere to the activated KCs in the early-phase of IRI. This adherence to KCs disturbs hepatic microcirculation and aggravates hepatic IRI. These effects are mediated by the PAF released from activated KCs [67]. Furthermore, deletion of the serum complement attenuates KC-induced oxidative stress. Complement-depleted animals have decreased the accumulation of neutrophil [68-70]. Interestingly, it was found that macrophage extracellular trap was increased in co-cultured experiment subjected to IRI. Moreover, macrophage extracellular trap aggravated ferroptosis, thereby causing an increasing post-ischemia liver damage [71].

However, the effects of KCs in other studies are opposite. In most studies, the role of KCs was investigated at the tissue level but not at cell type-specific level. Tissue resident macrophages, derived from yolk sac, play an important role in the maintenance of tissue homeostasis [72,73]. Previously, it was found that the activation of immune response by IR was associated with the necrotic depletion of KCs. During post-ischemia, KCs conferred anti-inflammatory and anti-lethality effect, indicating that KCs can be a novel mechanism against liver IRI, such as RIP-1-dependent necrosis [74]. In addition, fully mature F4/80^hi^ GATA6^+^ peritoneal cavity macrophages were reported to be recruited to the injury site via the mesothelium following liver injury. These cells acquired an activated phenotype and dismantle nuclei of necrotic cells to promote full revascularization of the injury site [75].

Heme oxygenase-1 (HO-1), a rate-limiting enzyme mainly released by KCs, emerges the anti-oxidative and anti-inflammatory functions. It has been reported that treatment with HO-1 inducer, Copp, decreased the levels of many inflammatory factors [76]. HO-1-SIRT1-p53 complex downregulated KCs recruitment thereby alleviated IRI [77]. In addition, a recent study has found that the activation of the NRF2/HO-1 pathway suppressed the NLRP3 inflammasome via enhancing KC autophagy, hence alleviated hepatic IRI [78]. Although KCs can polarize to M1 or M2, they usually polarize to M1 type after hepatic IRI, which aggravates hepatic IRI [79]. Activation of M2 macrophages has been reported to counteract the pro-inflammatory effects of M1 when activated, which inhibits pro-inflammatory signaling [80]. A previous study has demonstrated that SS-31, a mitochondrial-targeted antioxidant peptide, directly decreases ROS production and regulates signal transducer and activator of transcription 1/3 (STAT1/STAT3) signaling in macrophages, causing M2 polarization phenotype. The peptide also decreased the production of proinflammation cytokines, thereby mitigating inflammatory response in the liver [81].

### 4.3 T lymphocyte

T lymphocytes are derived from bone marrow pluripotent stem cells, which are a major component of lymphocytes. Mature T-lymphocytes are located in thymus-dependent areas of peripheral immune organs and involved in cellular immunity and immune regulation. In 1997, it was reported for the first time that T-lymphocytes are increased rapidly in post-ischemic liver, and it is the CD4^+^ T-cells but not CD8^+^ T-cells that accumulate in the liver 1 h after IRI [82].

An antigen-independent mechanism of T-lymphocyte activation following RANTES stimulation has been found to initiate the gathering of T-lymphocyte directly via a G-protein-coupled pathway [83]. Additionally, the CD154-CD40 T-cells co-stimulation pathway has been identified as an effective driver of T-lymphocyte accumulation and activation [84]. IL-17, released by T-lymphocytes, is associated with the recruitment of neutrophils. IL-17 also contributes to CXC chemokine secretion through other cells, including endothelial cells, fibroblasts, epithelial cells, and osteoblasts. Mice with CD4-konckout and treatment with anti-IL-17 antibodies showed reduced expression of macrophage inflammatory protein 2 (MIP-2) [85,86]. About 30% of recruited CD4^+^ T-cells are located in hepatic sinusoids, where they enhance platelet-adherence and neutrophil-recruitment, causing micro-vascular injury and hepatocyte cell death [87,88].

As early as in 1995, lymphocytes were reported to exert pro-inflammatory or anti-inflammatory effects by producing IFN-γ or IL-4, respectively [89]. IFN-γ triggers early inflammatory responses and KCs activation, but IL-4 suppresses the inflammatory response. Additionally, T-cell immunoglobulin mucin (TIM) family members may downregulate IR-triggered hepatic injury and cytokine/chemokine programs [90]. Indeed, interaction between TIM-3 and its galectin-9 (Gal-9) ligand inhibits Th1-mediated auto/allo-immune responses. Specifically, it can promote peripheral immune tolerance by readjusting macrophage activation and supporting hepatic homeostasis [91,92].

### 4.4 Neutrophils

Neutrophils, as innate immune cells, can identify inflammatory sites and eliminate microorganisms or damaging cells. It is inflammatory response overreaction that causing liver IRI. Some studies suggest that neutrophils have a pivotal role mainly in the late period of liver IRI [93,94].

Although hepatocytes generate ROS during the initial period of hepatic injury, it is ROS released by neutrophils that causes the most severe injury to hepatocytes, leading to mitochondrial permeability transition or mitochondrial dysfunction with calcium accumulation [95]. Once recruited to liver, neutrophils can express cell-surface adhesion molecules, such as P-selectin, L-selectin, and β2-integrins (CD11b/CD18) which bind to hepatocytes through the intercellular adhesion molecule-1 (ICAM-1) and vascular adhesion molecule-1 (VCAM-1) on LSECs [96,97]. It has been suggested that high expression of adhesion molecules, especially P-selectin, increases neutrophils production and subsequent liver damage [98]. In one study, recombinant P-selection glycoprotein ligand immunoglobulin (rPSGL-Ig), as a glycoprotein that binds P-selectin, was found to inhibit neutrophil adhesion. Moreover, desferriexochelin 772SM (D-Exo) enhanced the capacity of rPSGL-Ig to better protect against liver IRI [99]. Degranulation of activated neutrophils may release numerous proteases, including cathepsin G, elastase, heparinase, and hydrolytic enzymes [100]. It has been found that treatments with protease inhibitors can attenuate hepatic damage, thus the ONO-5046 may become an effective target for liver IRI prevention [101].

### 4.5 Liver sinusoidal endothelial cell

Liver sinusoidal endothelial cells (LSECs) account for up to 70% of hepatic non-parenchymal cells. LSECs form the vascular wall of hepatic sinusoid but do not have an organized basal membrane. The cytoplasm of these flattened cells contains clusters called sieve plates, which make the hepatic microvascular endothelium discontinuous. Reports have suggested that LSECs confer protection against inflammation and regulate vascular homeostasis, vascular tone, and toxicant clearance [102]. On the other hand, hepatic sinusoid has been found to be susceptible to IRI. Injury to LSECs is more serious compared to injury to hepatocytes [103]. LSECs are the main source of IL-33 in normal liver, although necrotic cells also release IL-33 which signals tissue damage. IL-33 can activate cyclin D1, p38, MAPK and Bcl-2, thereby protect against hepatic injury and suppress inflammatory responses [104-106].

However, LSECs have been shown to exert detrimental effects. For example, the elevation of ICAM-1 in LSECs was reported to aggravate hepatic IRI, leading to the aggregation and adherence of neutrophils and platelets to LSEC, which stagnate the hepatic sinus microcirculation [107]. Other studies have shown that LSECs release NO and ET, which help to balance of hepatic microcirculation. Increased levels of ET and thromboxane (TXA2) after IR can trigger contraction of the sinusoidal lumen and HSCs, hence exacerbate the injury [108].

## 5. Metabolic compartment

**Figure 3.**
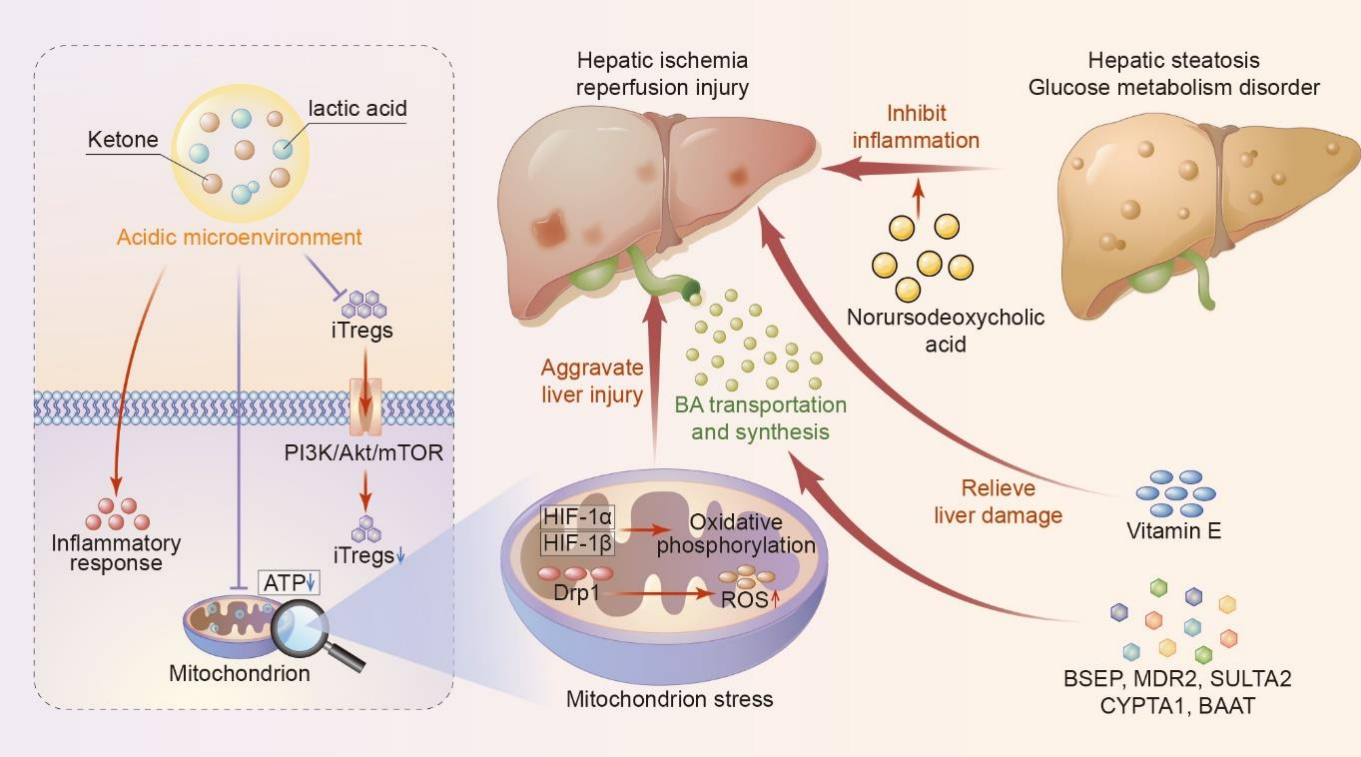
**Metabolic compartments regulate the process of hepatic ischemia/reperfusion injury**. A summary of metabolic components of the liver microenvironment during phase of ischemia/reperfusion injury is manifested. iTregs, inducible regulatory T cells; ATP, adenosine triphosphate; BA, bile acid; Drp1, dynamin-related protein 1; HIF-1α, hypoxia-inducible factor-1.

The metabolic compartments are also generally thought to influence the degree of hepatic IRI [109]. Assessment of metabolic variation over time can reveal the pathophysiological state of hepatic IRI and is important to the optimal choice of treatments (Fig. 3).

### 5.1 Metabolic acidosis

Acidic microenvironment, as a result of accumulation of acidic substances such as ketone bodies and lactic acid, promotes hepatocyte injury associated with IRI. High levels of lactate act as Damage-Associated Molecular Patterns (DAMPs) thus promoting inflammatory response at the reperfusion phase [110]. Furthermore, intracellular acidosis causes imbalance of protein turnover leading to enzymatic inhibition and vital protein destruction as well as blocking ATP reserve reconstitution after reperfusion [111,112].

Notably, iTregs are different from naive T-cells in the peripheral environment in that they promote the recovery of liver function after IRI. Acidic microenvironment inhibits the generation and function of CD4^+^CD25^+^Foxp3^+^ iTregs through PI3K/AKT/mTOR signaling [113,114]. Several studies have explored the role of acid-base homeostasis in maintaining normal cellular response and immune system homeostasis. Furthermore, acidic microenvironment triggers upregulation of NO synthase in macrophages, accumulation of neutrophils, deactivation of the cytoplasmic- and membrane-associated enzyme, as well as downregulates synthesis of cAMP, proteins, and DNA [115,116]. Furthermore, Golse *et al.*reported that arterial lactate concentration at the end of LT (LCEOT) ≥5 mmol/L is an effective predictor of early graft outcomes [117]. Therefore, acidic microenvironment plays an important role in the progression of liver IRI, and lactate clearance can be used to alleviate liver IRI.

### 5.2 Glucose and fatty acid metabolism

One study have demonstrated that the availability of glycolytic substrates maintains ATP and promotes functional recovery during reperfusion phase [118]. Given this, the low pool of glycogen in IR causes more rapid ATP depletion, as well as alterations in tissue antioxidant defenses and dysregulation of mitochondrial function [119]. However, a different study reported that low pool of glycogen can reduce KCs phagocytosis and the generation of TNF-α thus improving organ viability and survival during long periods of ischemia [120]. Therefore, a beneficial effect of high glycogen content is mainly observed during short ischemia, whereas low metabolic reserves are preferentially needed in the process of long ischemia [102].

Glycogen synthase kinase 3 (GSK3) is a ubiquitous serine/threonine kinase, involved in regulation of glycogen synthesis [121]. Notably, inhibition of GSK3 in liver IRI models ameliorates liver damage via an IL-10-mediated immune regulatory mechanism resulting in reduced serum ALT levels and complete lobular architecture. Inactivation of GSK3 in liver IR is a self-regulatory mechanism in liver homeostasis that occurs through activation of PI3K [122,123]. In addition, Zhou *et al.* [124] demonstrated that GSK3β promotes activation of macrophage inflammation by suppressing the AMP-activation protein kinase and inducing the novel innate immune negative regulator SHP. Moreover, TGR5 is implicated in the regulation of energy homeostasis and glucose metabolism [125]. Although their roles in modulation of innate immune activation in liver IRI have been widely explored, their roles in regulating liver metabolism to influence hepatic IRI have not been fully elucidated.

Selzner *et al.* [126] reported that steatotic livers are vulnerable to hepatocyte damage and may be associated with poor tolerance to IRI. Steatotic livers produce less ATP compared with non-steatotic livers during the hepatic IRI, due to upregulation of the mitochondrial uncoupling protein 2 expression [127]. Additionally, hepatocytes with fatty infiltration have been found to develop massive necrosis after IRI, whereas apoptosis is mainly observed in non-steatotic livers after IRI [128]. In addition, steatotic livers exhibit lower expression of inositol-requiring enzyme 1 and PKR-like endoplasmic reticulum kinase, which may increase the risk of steatotic livers to IRI [129]. Elke *et al.* [130] revealed that steatosis exacerbates early IRI by enhancing effector immune cell infiltration, including higher mRNA expression of CXCL-1 and CD3.

### 5.3 Metabolic dysregulation of mitochondria

Mitochondria is a major target in ischemia injury, and dysregulation of mitochondria homeostasis and cellular energetics aggravate liver damage following ischemia injury. ROS released by the mitochondria during ischemia injury beyond antioxidant capacities promotes cellular DNA damage, calcium overload and mitochondrial lipid peroxidation. This leads to the release of cytochrome c and cellular damage [131,132].

Intracellular trafficking of mitochondria plays an important role in meeting local metabolic demands as well as self-renewal of the organ. Tunneling nanotubes (TNTs) mediate intercellular transfer of mitochondria. Notably, inhibitors of TNT were found to decrease mitochondrial intercellular transfer, thus alleviating the ischemia injury [133]. Given these results, use of stem or progenitor cells as a vehicle for normal mitochondrial diversion to cells with damaged mitochondria as a result of ischemia injury presents high therapeutic potential [134].

Hypoxia-inducible factor-1 (HIF-1α) significantly accumulated during ischemia injury, it binds to the β submit and is translocated to the nucleus to promote transcription of other genes. Most of these genes could be implicated in the glycolytic pathway and causes a switch in the production of energy from oxidative phosphorylation to glycolysis, thus exacerbating hypoxia in organs [135]. Moreover, dynamin-related protein 1 (Drp1) modulates the morphology of mitochondria and inhibits protective mitophagy by upregulating expression of mito-Clec16a. Moreover, Drp1 mediates metabolic disorders and decreases the levels of mitochondrial glutathione thus impairing free radical scavenging, resulting in further increase in ROS levels [136].

### 5.4 Role of metabolites in liver IRI

Several metabolites have been reported to associated with adverse liver-related events, as well as liver IRI. Some studies reported that genes associated with bile acid (BA) transportation and synthesis (i.e., BSEP, MDR2, SULTA2, CYP7A1 and BAAT), as well as nuclear factors implicated in regulation hepatic metabolism (i.e., SREBF1 and FXR), are tied with progression of hepatic IRI [137-139]. BA is an important signaling molecules with pleiotropic effects on liver physiology. High serum levels of taurochenodeoxycholate, which is a complex formed from conjugation of the BA chenodeoxycholate with taurine, is associated with a rapid liver-related adverse events. On the contrary, norursodeoxycholic acid, as a secondary bile acid, exhibits positive effects on the liver histology [140]. These findings indicate that targeting different bile acids can alleviate liver IRI.

A previous retrospective single-center cohort study, which included 187 participants, reported that vitamin E, primary bile acid and serotonin are associated with occurrence of future liver-related events [141]. A double blind randomized, and placebo-controlled trial was conducted previously whereby patients received three infusions containing vitamin E and the results showed significant improvement for ALT, AST and lactate dehydrogenase (LDH) levels after surgery [142].

Serotonin can be produced by cholangiocytes and stellate cells in the liver. Moreover, it also can be produced in the gut then it is metabolically transformed to 5-hydroxyindoleacetic (5-HIAA) in the liver [143]. Serotonin significantly enhances human megakaryocytes (MKs) growth through 5-HT_2B_R with subsequent activation of p-Erk1/2, which induces cytoskeleton reorganization and subsequent proplatelet formation [144]. Platelets can induce opposite effects by causing ischemia liver injury as well as promote subsequent tissue repair process. Nocito *et al*. [145] reported that platelets promote tissue repair and liver regeneration after normothermic hepatic ischemia in mice. Meanwhile, platelet-derived serotonin stimulates the proliferation of liver.

## 6. Remedies of hepatic IRI

Despite the tremendous efforts to develop therapies for IRI, the available treatments for IRI are not effective in all subsets of patients. Numerous studies have explored therapeutic efficacy of amino acid drugs (such as glycine and N-acetylcysteine), oxygen radical reducing drugs (including antioxidants, nitric oxide and carbon monoxide) and anti-inflammatory drugs (prostacyclin, atropine, and glucocorticoids) in treatment of IRI. Several molecular factors and genes have also been investigated as potential treatment targets, such as interferon regulatory factors, IL-10, programmed death factor, and bcl-6 [146]. A summary of these pharmacological and gene-based therapeutic targets is provided inTable 1.

**Table 1:** Pharmacological/Gene therapy.

Time	Strategies and Description	Species	Ischemic time	Effect	Result
**2008** [170]	PPAR-α agonists and adiponectin siRNA	Rat	60 min	MAPK expression and adiponectin accumulation↓	Oxidative and hepatic injury↓
**2008** [171]	Allopurinol and apocynin (inhibitor of XOD and NADPH oxidase)	Mice	30 min	Generation of superoxide anions↓	Hepatic injury↓
**2008** [172]	Ascorbate (scavenger of ROS)	Rat	30 min	Apoptosis of KCs↓	Hepatic injury↓
**2008** [173]	Captopril (Ang 2 blockers)	Rat	60 min	BK generation and PPAR-γ ↑	Hepatic injury↓
**2008** [174]	Tetrandine (scavenge ROS and inhibit lipid peroxidation)	Mice	90 min	Neutrophil accumulation, TNF-α and MDA↓ SOD↑	Liver edema and hepatic injury↓
**2009** [175] [176]	Mutation of TLR4 or TLR4 knockout	Mice	60 min	Release of pro-inflammatory cytokines and neutrophil infiltration↓	Hepatic injury and damage of LSECs↓
**2010** [177]	Carbon monoxide-releasing molecule-2 (CORM-2)	Rat	60 min	Neutrophil infiltration, TNF-α, IL-6, ICAM-1↓ Bcl-2↑	Hepatic injury and levels of apoptosis↓
**2010** [178]	Metron factor-1 (MF-1)	Rat	90 min	Oxygen free radicals↓ NO synthesis and survival↑	Hepatic injury and oxidative stress↓
**2010** [179]	Sirolimus (immunossupressant drug)	Rat	60 min	Tissue myeloperoxidase and neutrophil infiltration↓	Hepatic injury and liver cell apoptosis↓
**2011** [180]	Atorvastatin (HMG-CoA reductase inhibitor)	Mice	60 min	STAR overexpression and mGSH depletion↓	Hepatic injury and oxidative stress↓
**2011** [181]	n-3 PUFA (polyunsaturated fatty acid)	Rat	60 min	NF-κB, TNF-α and IL-1β↓	Hepatic injury and oxidative stress↓
**2011** [182]	rPSGL-Ig (selectin antagonist)	Human	60min	IL-10↑	Hepatic injury and oxidative stress↓
**2012** [183]	ABC294640 (selective inhibitor of sphingosine kinase-2)	Mice	60 min	S1P, neutrophil infiltration, NO synthase, NF-κB and TNF-α↓	Hepatocyte death and hepatic injury↓
**2012** [184]	Fasudil (a Rho-kinase inhibitor)	Rat	30 min	HSC activation, endothelin 1 and portal perfusion pressure↓	Hepatic injury and hepatic susceptibility↓
**2012** [185]	Nilotinib (tyrosine kinase inhibitor and against JNK and p38 in vitro)	Mice	60 min	Recruitment of inflammatory monocytes, IL-1β, IL-6, MCP-1, MIP-2, JNK and p38 MAPK↓	Hepatocyte apoptosis and hepatic injury↓
**2012** [186]	Deletion of FGL2/Fibroleukin (transgenic)	Mice	60 min	Hepatocyte and LSEC protection	Hepatic injury and IRI cascade↓
**2013** [187]	RMT1-10 (TIM-1 blocker)	Mice	20 h	Neutrophil and macrophage infiltration/activation, NF-κB and IFN-γ↓ IL-10, IL-22, Bcl-2↑	Hepatic injury and oxidative stress↓
**2013** [188]	Simvastatin (immunossupressant drug)	Mice	16 h	Autophagy induction, LSEC injury↓ NO↑	Liver damage, oxidative stress and endothelial dysfunction↓
**2014** [189]	rMnSOD (antioxidant)	Mice	20 min	Accumulation of superoxide anion and inflammation↓ NO↑	Hepatic injury and oxidative stress↓
**2015** [190] [191]	Knockout of IRF9	Mice	60 min	Serum ALT/AST, immune cell infiltration and levels of inflammatory cytokines ↓	Hepatic injury and hepatocyte apoptosis↓
**2016** [192]	Total flavonoids (TFs)	Rat	60 min	MPO, LDH, MDA, IL-6, TNF-α and IL-β↓ SOD and GSH-Px↑	Improve liver histopathology and ultrastructure
**2016** [193]	Overactivation of Nrf2-ARE	Mice	60 min	IL-6, IL-1β and levels of 8-isoprostanes↓	Hepatocellular damage, necrosis, apoptosis and oxidative stress↓
**2017** [194]	Extracellular vesicles from mesenchymal stem cell (MSC-EV)	Murine	90 min	NF-κB and IL-6↓ Expression of NACHT, LLR and PYD domains-containing protein 12↑	Hepatic caspase 3-positive and apoptotic cells↓
**2017** [195]	Inhibition of RAP1/KC/NLRP3 inflammasomes	Mice and human	45 min	Activation of NLRP3 and levels of ALT and ALT↓	Hepatic protection
**2018** [196]	Knockout of CARD6	Mice	60 min	NF-κB, JNK, p38 and inflammatory chemokines↓	Hepatic injury and liver cell death↓
**2019** [197]	Salicylate acetyl-3-aminoethyl salicylic acid (ac3AESA)	Murine	60 min	Activation of KC, IL-6, TNF-α, IL-β, CXCL2 and CXCL8↓	Hepatic injury and allograft damage↓
**2019** [198]	Omeprazole (buffer the acid microenvironment)	Mice and human	60 min	Function of CD4CD25Foxp3 iTregs↑	Hepatic injury↓
**2020** [199]	PINK1 (mediate mitophagy)	Mice	60 min	ROS production, NLRP3, and KC-mediated inflammation↓	Hepatic injury and mitochondrial dysfunction↓
**2020** [200]	Inhibition of miR-450b-5p	Mice	60 min	CRYAB and M2 polarization↑ NF-κB↓	Hepatic protection
**2021** [201]	Overexpression of miR122	Mice and human	60 min	PHD1↓ HIF1α expression↑	Hepatic ischemia tolerance↑
**2021** [202]	Inject rhMANF	Mice and human	90 min	Activated ATF4/CHOP and JNK/c-JUN/CHOP pathways↓	UPR injury and hepatocellular damage↓

PPAR-α, peroxisome proliferators-activated receptor-α; PPAR-γ, peroxisome proliferators-activated receptor-γ; TLR4, Toll like receptor 4; TNF-α, tumor necrosis factor α; NLRP3, nucleotide-binding oligomerization domain-like receptor family pyrin domain containing 3; IL-1β, interleukin 1β; HMGB1, high-mobility group box 1; IAC, inflammation associated cytokine; IL-1, interleukin 1; IL-6, interleukin 6; IL-10, interleukin 10; IL-22, interleukin 22; NF-κB, nuclear factor κB; ICAM-1, intercellular adhesion molecule-1; Bcl-2/Bcl-x, B cell lymphoma 2/x; MIP-2, macrophage inflammatory protein 2; JNK, N terminal kinase; IFN-γ, interferon γ; ATF4, activating transcription factor 4; CHOP, C/EBP homologous protein; LDH, Lactate dehydrogenase; MDA, malondialdehyde; SOD, superoxide dismutase; GSH-Px, se-dependent enzyme glutathione peroxidase.

Further clinical trials should be conducted to develop and test effective interventions for hepatic IRI. Evidence from animal experiments have shown that curcumin treatment alleviates the negative effects of IRI in different organs including brain, heart, kidney, intestine, ovary, testis and liver [147-154]. Therapeutic effects of curcumin are mediated by mechanisms such as anti-oxidative stress, anti-inflammation and reduction of adhesion molecules thus ameliorating hepatic IRI. Curcumin has been reported to suppress oxidative stress by upregulating expression of antioxidant enzymes and inhibiting production of ROS [155]. Notably, curcumin has a strong intrinsic activity, thus curcumin is applied in treatment of various diseases. However, some studies also have reported limitations associated with bioavailability of curcumin such as limited tissue distribution, low serum levels, short half-life and apparent rapid metabolism [156]. Adjuvants which can bypass the metabolic pathways of curcumin, are revealed to be one of the major therapies used to improve its bioavailability. For instance, liposomes, micelles, nanoparticles and phospholipid complexes are used to improve the bioavailability of curcumin [157,158]. In addition, various extracts or secretions from traditional Chinese medicinal herbs may potentially inhibit oxidative stress and inflammation. For example, resveratrol and pterostilbene exhibit anti-cell proliferation, anti-oxidative stress and anti-inflammation effects [159]. However, further animal and clinical studies are needed to further verify these findings.

Mesenchymal stem cells (MSCs) have unique immunomodulatory properties. MSCs are invaluable cell types used for repair of tissue or organ damage. MSCs have been reported to suppress the infiltration of inflammatory cytokines and promote expression of anti-inflammatory cytokines [160]. Kharaziha *et al.* [161] and Mohamadnejad *et al.* [162] carried out successful trials which indicated that transplantation of autologous MSCs significantly improves liver function in IRI patients. In addition, MSCs are effective delivery vehicles characterized by injury tropism [163]. A previous study reported that engineered human induced pluripotent stem cells (hiPSC-MSCs) delivering GPx3 significantly suppresses senescence of liver and then alleviates hepatic IRI [164]. Therefore, therapeutic properties of MSCs on IRI have high potential for treatment of hepatic IRI.

Gene therapy is novel strategy for treatment of patients by modulating gene expression through approaches such as knockout, knockdown, correction or knock-in, thus it has high potential therapy for treatment of various diseases [165]. Knockout of CARD6 exhibits beneficial effects against myocardial IRI, by modulating apoptosis signal-regulating kinase 1 (ASK1) and several other signaling pathways. This implies that inhibition of ASK1 is an effective strategy for the treatment of hepatic IRI. Viral-based methods present an effective choice for the delivery of genes in gene therapies, with adeno-associated virus (AAV) being the most promising viral vector [166]. However, limitations such as packaging capacity (<4.7 kb), safety concern correlated to immunogenicity, and high cost associated with AAV restrain application of AAVs in gene therapy [167]. In light of this, studies are exploring chemical-based methods, such as polymer-based vectors as alternatives, owing to their low cost, high tunability and immune-compatibility [168]. For example, Reineke *et al.* [169] designed a new class of carbohydrate-based polymers and referred them as poly(glycoamidoamine)s (PGAAs), which are effective and biocompatible transfection reagents.

## 7. Conclusion and prospect

Numerous clinical and animal experiments have been conducted to explore molecular mechanisms associated with hepatic IRI and the findings show high potential in development of therapies for hepatic IRI. However, the complex interactions between hepatic microenvironment and IRI have not been fully elucidated thus limiting design of effective regimens for hepatic IRI patients. Possible mechanisms of liver IRI, including the interaction of various immune cells, effects of metabolites on IRI progression and the role of mitochondrial in liver IRI have been summarized in the present study.

Findings from pre-clinical studies indicate that several therapies are effective, however, results from clinical trials present low efficacy. Mechanisms of hepatic IRI vary with experimental conditions, such as period of ischemia (minutes to weeks), extension of ischemia (partial or complete), type of ischemia (cold or warm) and targeted immune cells. Therefore, findings from animal experiments cannot be directly translated to the clinical management of IRI. Furthermore, potential application of basic research knowledge should be taken into consideration owing to the extended-criteria or even increasingly complex situations of exact conditions of patients.

Furthermore, the possible cell therapy and gene therapy which are promising strategies for IRI treatment are explored from a clinical/translational perspective in the current review. However, the cross-talk between the hepatic microenvironment and the processes of IRI have not been fully explored, thus further studies should be conducted to fully elucidate this cross-talk. Studies should explore the pathogenesis of hepatic IRI to provide a basis for designing therapeutic strategies to ameliorate hepatic IRI, or even cure the disease.
